# Lacidophilin tablets alleviate constipation through regulation of intestinal microflora by promoting the colonization of *Akkermansia sps*

**DOI:** 10.1038/s41598-024-57732-5

**Published:** 2024-03-26

**Authors:** Denglong Sun, Jingting Yu, Yang Zhan, Xiaoying Cheng, Jingwen Zhang, Yingmeng Li, Qiong Li, Yanxia Xiong, Wenjun Liu

**Affiliations:** 1 Research and Development Department, Jiangzhong Pharmaceutical Co., Ltd., No. 1899 Meiling Road, Nanchang, 330103 Jiangxi Province People’s Republic of China; 2 National Key Laboratory for the Modernization of Classical and Famous Prescriptions of Chinese Medicine, No. 788 Huoju Avenue, Gaoxin Dev District, Nanchang, 330096 People’s Republic of China; 3 Department of Food Nutrition and Safety, College of Pharmacy, Jiangxi University of Chinese Medicine, Nanchang, 330004 Jiangxi Province China

**Keywords:** Dysbiosis, Constipation

## Abstract

Constipation is a major health problem worldwide that requires effective and safe treatment options. Increasing evidence indicates that disturbances in gut microbiota may be a risk factor for constipation. Administration of lacidophilin tablets shows promising therapeutic potential in the treatment of inflammatory bowel disease owing to their immunomodulatory properties and regulation of the gut microbiota. The focus of this study was on investigating the ability of lacidophilin tablets to relieve constipation by modulating the gut microbiome. Rats with loperamide hydrochloride induced constipation were treated with lacidophilin tablets via intragastric administration for ten days. The laxative effect of lacidophilin tablets was then evaluated by investigating the regulation of intestinal microflora and the possible underlying molecular mechanism. Our results reveal that treatment with lacidophilin tablets increased the intestinal advancement rate, fecal moisture content, and colonic AQP3 protein expression. It also improved colonic microflora structure in the colonic contents of model rats mainly by increasing *Akkermansia muciniphila* and decreasing *Clostridium_sensu_stricto_1*. Transcriptome analysis indicated that treatment with lacidophilin tablets maintains the immune response in the intestine and promotes recovery of the intestinal mechanical barrier in the constipation model. Our study shows that lacidophilin tablets improve constipation, possibly by promoting *Akkermansia* colonization and by modulating the intestinal immune response.

## Introduction

Constipation is a prevalent gastrointestinal disorder with a high worldwide incidence^1^. It has a rapidly increasing incidence rate, and is associated with aging, dietary habits, psychosocial factors, ecological changes, and accelerated modern lifestyles^2,3^. It is characterized by reduced frequency of bowel movements, hard stools, excessive straining during defecation, anal bleeding, and rectal stimulation^4^. Although constipation is usually dismissed as a mere nuisance, it adversely affects the quality of life and poses a considerable economic and medical burden^5^. Constipation has also been linked to an increased risk of gastrointestinal diseases including irritable bowel syndrome and colorectal cancer^6,7^. Currently, laxatives are generally used to alleviate constipation. However, drug intervention can easily lead to drug dependence and relapse upon discontinuation and may also cause adverse reactions such as dehydration and dizziness^8,9^. Hence, there is a serious need for the development of effective treatments for constipation.

Gut microbiota play a crucial role in gut motility. An increasing number of reports suggest that disruption of gut microbiota may be a risk factor for constipation^10,11^. Germ-free mice display longer gastric emptying and intestinal transit times versus normal mice. However, when *Lactobacillus acidophilus*, *Bifidobacterium bifidum*, and *Clostridium tabificum* colonize the gut, the intestinal transit time is shortened, whereas colonization with *Micrococcus luteus* and *Escherichia coli* has the opposite effect^12^. By investigating the characteristics of gut microbiota of constipated and healthy individuals, it was found that the number of beneficial bacteria (such as *Bifidobacterium* and *Lactobacillus*) in the gut microbiota of constipated patients was significantly reduced, and the number of Proteobacteria (mainly *Enterobacter*, *Enterococcus*, and *Fusobacterium*) increased significantly^13,14^. In addition, dysbiosis of gut microbiota can affect the composition of their metabolites, such as short-chain fatty acids (SCFAs) and tryptophan breakdown products^15^. SCFAs can inhibit inflammatory responses, stimulate secretion of gut regulatory peptides, and promote gut motility^16^. 5-hydroxytryptamine (5-HT), produced by tryptophan in the gut microbiota, has been shown to increase gut motility and relieve constipation^17^. Therefore, maintaining the composition and function of gut microbiota is an effective strategy for preventing constipation.

Lacidophilin tablets (LP) are a product obtained by fermenting milk with *Lactobacillus acidophilus*. After fermentation, the cells and fermentation broth are spray-dried to produce LP. Lacidophilin thus mainly contains *Lactobacillus acidophilus* cells and their metabolites from breakdown of milk. LP has been widely used in clinical practice for the treatment of enteritis, indigestion, and abnormal intestinal fermentation. In addition, our previous research confirmed that LP regulates gut microbiota^18^. However, there is a lack of effective evidence to prove whether LP can improve constipation by regulating gut microbiota in the context of constipation.

The aim of the present study was to uncover the effects of LP in the treatment of constipation. This was achieved using a loperamide-induced constipation model, followed by gut microbial analysis and intestinal transcriptome analysis to identify the molecular mechanism of LP in the treatment of constipation.

## Results

### LP improves defecation in the constipation model

**Figure 1:**
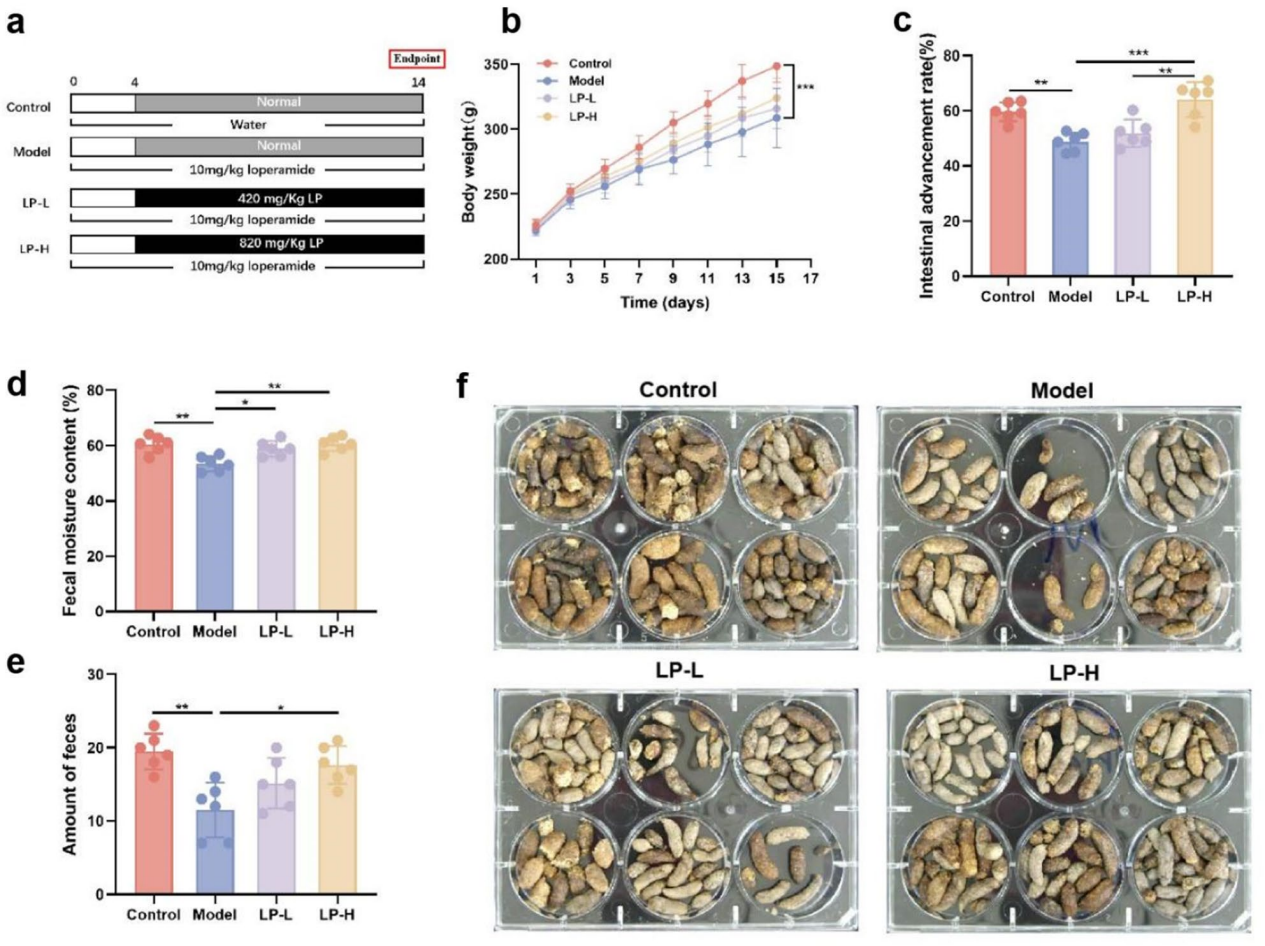
LP improves defecation in the rat constipation model. (**a**) Experimental layout of LP treatment in the constipation model. (**b**) Recording the body weight of rats. (**c**) Intestinal advancement rate. (**d**) Fecal moisture content. (**e**) Amount of feces. (**f**) Macroscopic assessment of feces. Values are expressed as mean ± SD. *p < 0.05, **p < 0.01, ***p < 0.001, n = 6.

Rat models of constipation were established as described in the “Methods” section and depicted in Fig. 1a. Constipation was induced by intragastric administration of 10 mg/kg loperamide once daily for 14 days in both model and experimental groups. Intragastric administration of LP was then performed as high doses (LP-H) or low doses of LP (LP-L). Compared to the model group, the LP-H and LP-L groups showed elevated body weights. However, the difference was not statistically significant (Fig. 1b). The intestinal advancement rate was lower in the model group than that in the LP-H group. Additionally, the LP-H group showed a significantly higher intestinal advancement rate than the LP-L group, yet LP-L also had limited therapeutic effects (Fig. 1c). Fecal samples were collected to estimate the fecal moisture content and amount of feces. We found that fecal moisture content was significantly higher in the LP-L and LP-H groups than in the model group (Fig. 1d). LP-H augmented the 24-h amount of feces compared to the model group. LP-L also had a similar effect on the 24-h amount of feces compared to the model group, although the difference was not significant (Fig. 1e). Representative feces from all groups are shown in Fig. 1f. Overall, these results confirm the successful establishment of the constipation models, and demonstrate that high doses of LP confer defecation benefits by enhancing the fecal water content and amount of feces.

### LP alterations of histopathological and cytological structure of colon

**Figure 2:**
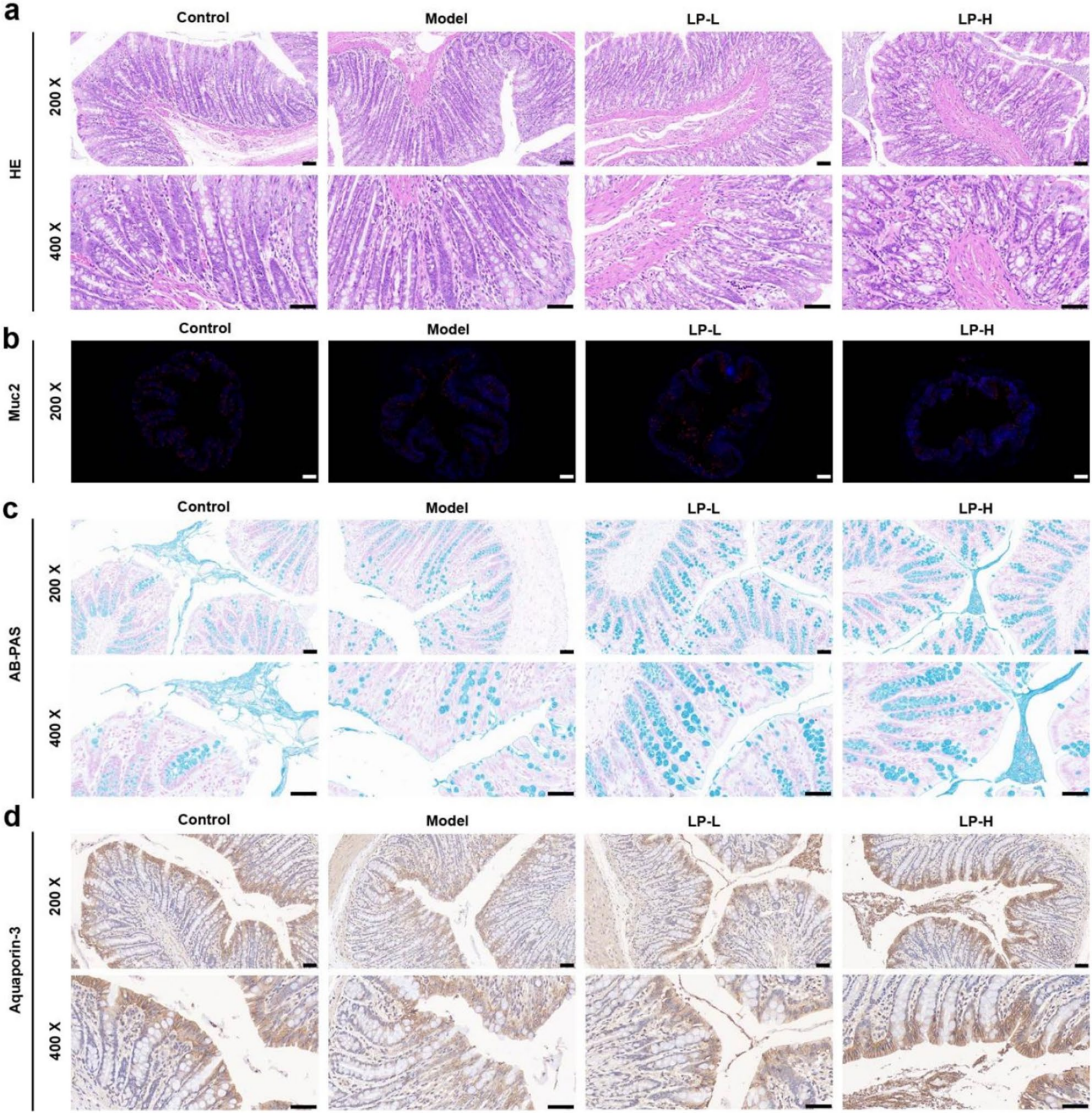
LP improves the colonic mucus barrier in the rat constipation model. (**a**) HE staining of colon tissue. (**b**) Muc2 staining of colon tissue. (**c**) AB-PAS staining of colon tissue. (**d**) Aquaporin-3 staining results of colon tissue.

To confirm the role of LP in modulating the histopathological and cytological structures of colon in the constipation model, we collected colon tissues to examine colon epithelial information using H&E staining, AB-PAS staining, and immunocytochemistry staining. As shown in Fig. 2a, the mucosal layer of the colon tissue was damaged and there was obvious thickening of the intestinal wall in the model group. The intestinal glands in the lamina propria were also dissolved and turned necrotic, along with disappearance of goblet cells and crypts, accompanied by severe inflammatory cell infiltration. The variation in goblet cell numbers, does it lead to an increase in Muc2 protein? Our immunofluorescence staining results for Muc2 indicate that LP-H enhances the expression of Muc2 protein (Fig. 2b). As compared to constipated rats, high doses of LP conferred obvious improvement to the structure of the colon in the LP-H group. AB-PAS staining indicated that LP-H increased the thickness of the colonic mucus layer (Fig. 2c). The expression of Aquaporin-3 in paraffin sections from all groups was detected using immunohistochemistry staining, and the results indicated that Aquaporin-3 expression in the model group was generally lower than that in the LP-H group (Fig. 2d). This further demonstrated that LP-H improves the histopathological and cytological features of the colon.

### Diversity of different gastrointestinal tract microbial communities arising from the LP

**Figure 3:**
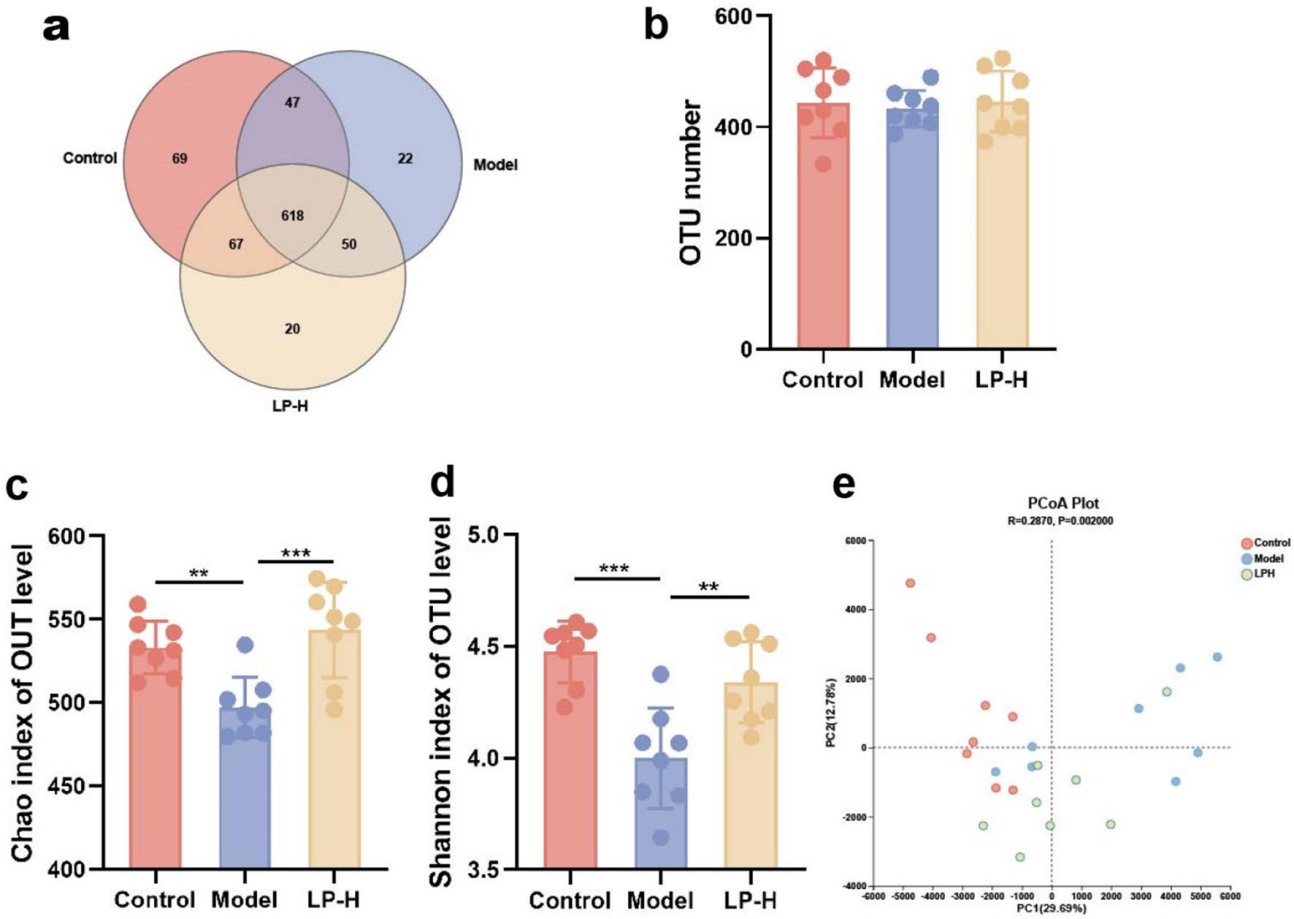
LP Improves the diversity of gut microbiota in constipated rats. (**a**) Venn diagram summary of the numbers of operational taxonomic units (OTUs) in the gut microbiota community of the control, model, and LP-H groups. (**b**) OTU number in different groups. (**c**,**d**) Alpha diversity indices including Chao and Shannon indices. (**e**) PCoA score plots in different groups; values are expressed as mean ± SD. *p < 0.05, **p < 0.01, ***p < 0.001, n = 8.

Gut microbiota play an essential role in intestinal function. This study explored the potential involvement of the gut microbiome in the alleviation of constipation with LP. We analyzed the gut microbiota community composition by 16S rRNA sequencing using the Illumina Hiseq platform in rats treated with LP after loperamide-induced constipation. The composition of fecal microbiota is shown in the Venn diagram (Fig. 3a). A total of 893 OUTs were identified in the three groups. As illustrated in Fig. 3b, OUT number of the LP group was higher than that of the model group, but the difference was not significant. α-diversity indices including Shannon (c) and Chao (d) indices are shown in Fig. 3. These two indices were higher in the control and LP groups than in the model group. Principal coordinate analysis with taxonomic information at the OUT level showed that the differences in the gastrointestinal tract microbiota among the three groups were significant (Fig. 3e). The LP group was found to be closely clustered with the control group, suggesting that LP treatment restores the composition of gut microbiota.

### Different gastrointestinal tract microbial composition is associated with LP treatment

**Figure 4:**
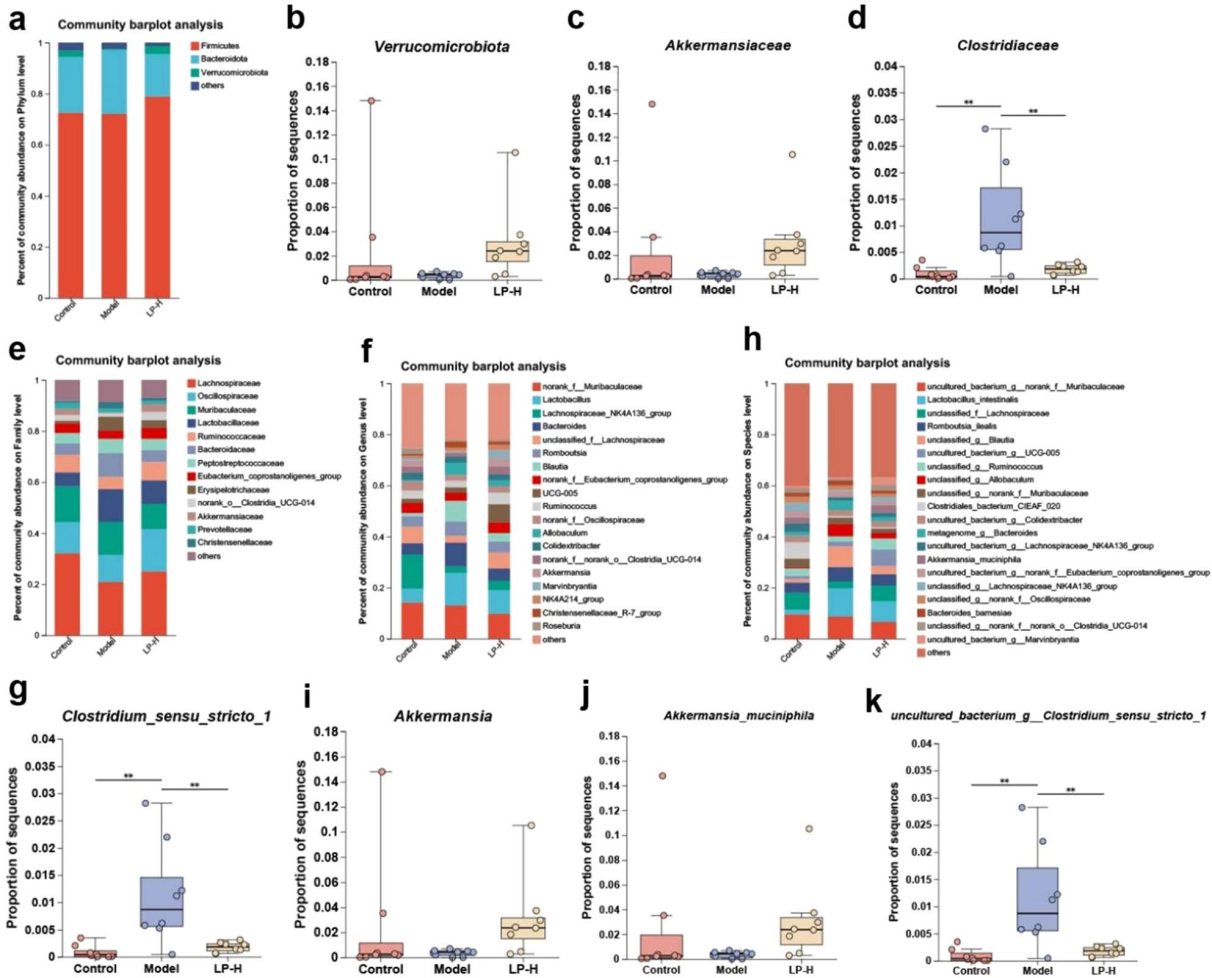
Composition of gut microbiota among different groups. (**a**) Phylum level. (**b**) Major differential microbiota at the phylum level. (**c**,**d**) Major differential microbiota at the family level. (**e**) Family level. (**f**) Genus level. (**g**,**i**) Major differential microbiota at the genus level. (**h**) Species level. (**j**,**k**) Major differential microbiota at the species level. Values are expressed as mean ± SD. *p < 0.05, **p < 0.01.

*Firmicutes, Bacteroidota* and *Verrucomicrobiota* were the dominant bacterial phyla in the feces (Fig. 4a). *Verrucomicrobiota* were significantly increased upon treatment with LP when compared to the model group (Fig. 4b). The dominant strains belonged to *Lachnospiraceae, Oscillospiraceae*, *Muribaculaceae, Ruminococcaceae* and *Catobacillaceae* families (Fig. 4e). There was a significant increase of *Akkermansiaceae* in the LP group when compared to the model group (Fig. 4c). Furthermore, loperamide induction significantly increased the abundance of the *Clostridiaceae* when compared to the control group, but LP treatment caused a reduction in the abundance of this strain (Fig. 4d). At the genus level, the results showed that *norank_f_Muribaculaceae*, *Lachnospiraceae_Nk4A136_group, unclassified_f_Lachnospiraceae, Lactobacillus* and *Bacteroides* were the dominant genera in all fecal samples (Fig. 4f). Compared to the model group, the relative abundance of *Clostriium_sensu_stricto_1* in the control and LP groups was significantly lower (Fig. 4g), whereas the relative abundance of *Akkermansia* was significantly higher (Fig. 4i). At the species level, *uncultured_bacterium_g_norank_f_Muribaculaceae, unclassified_f_Lachnospiraceae*, *Romboutsia ilealis*, *Clostridiales_bacterium_CIEAF_O2O* and *uncultured_bacterium_g_Lachnospiraceae_NK4A136*_group were dominant species (Fig. 4h). LP treatment significantly increased the abundance of *Akkermansia muciniphila* and significantly decreased the *uncultured_bacterium_g_Clostridium_sensu_stricto_1* (Fig. 4j,k).

### Transcriptome analysis of colon tissue

**Figure 5:**
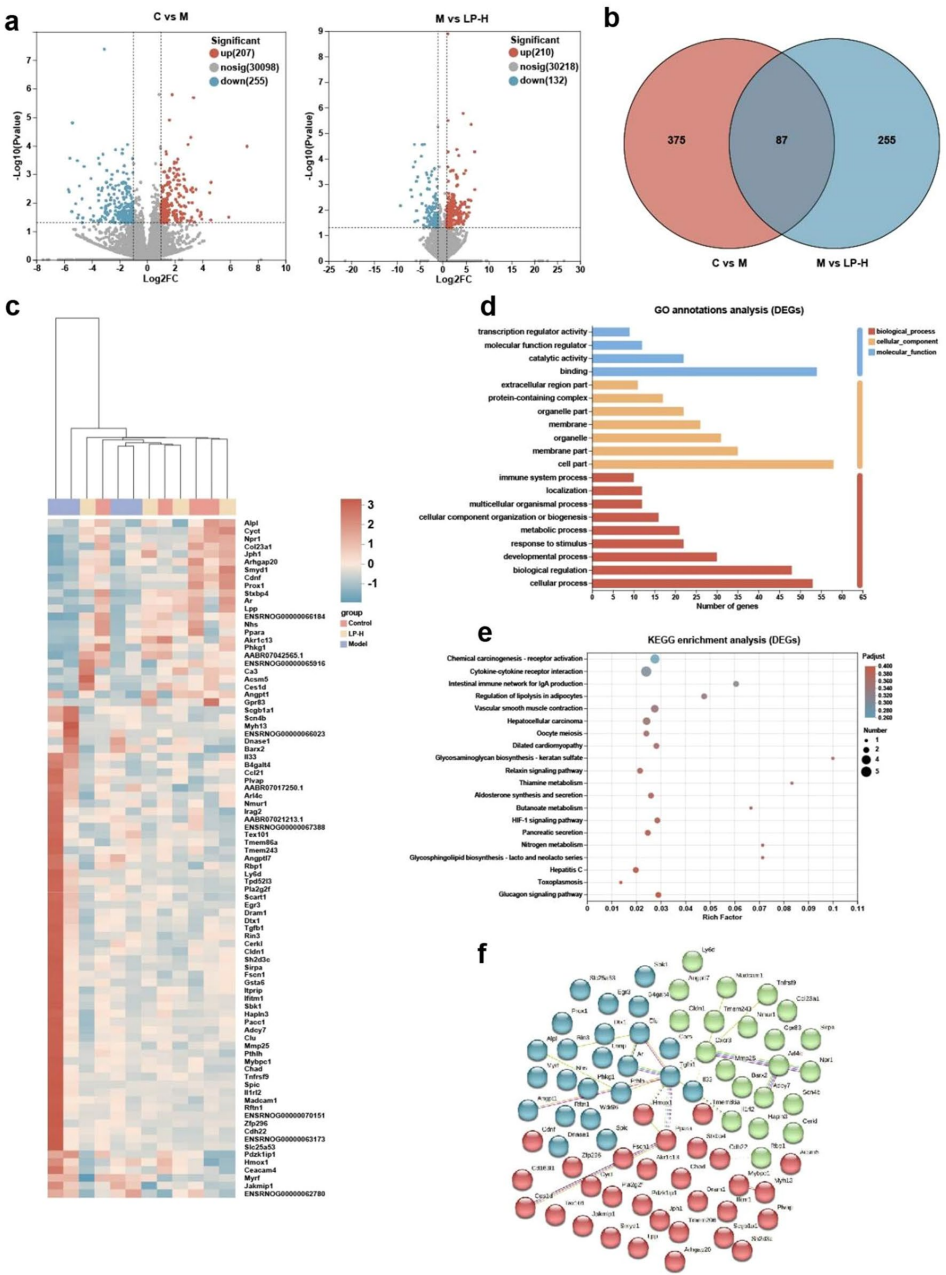
Results from RNA-sequencing of colon tissue among different groups. (**a**) Volcano plot of DEGs, red dots represent up-regulated genes and blue dots represent down-regulated genes. (**b**) Venn diagram summarizes the numbers of DEGs in different groups. (**c**) Heatmap of hierarchical clustering of DEGs. (**d**) Gene Ontology enrichment analysis of DEGs. (**e**) KEGG pathway enrichment analysis of DEGs. (**f**) PPI analysis of DEGs.

We performed RNA sequencing of colonic tissues in a constipation model to elucidate the molecular basis of the therapeutic mode of action of LP. Sequenced reads were mapped to the rat genome and significantly differentially expressed genes (DEGs) were defined by an adjusted *p*-value < 0.05, and fold change > 2. As shown in Fig. 5a, loperamide induced differential expression in 462 genes when compared to the control, including 207 upregulated and 255 downregulated genes; LP-H induced differential expression of 442 genes when compared to the model, including 210 upregulated and 132 downregulated genes. Venn analysis revealed differences in the expression of 87 genes through (Fig. 5b), indicating that these genes are potentially involved in the molecular mechanisms of the LP-H treatment model. Hierarchical clustering of simple and 87 DEGs showed differential expression between all groups (Fig. 5c). Gene Ontology (GO) analysis showed that the 87 DEGs were mostly enriched in annotations within the biological process and cellular component, especially in immune system process (Fig. 5d). These DEGs are related to antigen presentation, neutrophil activation, and chemokine-mediated signaling pathway, which are important for maintaining the mucosal barrier and preventing inflammation. The Kyoto Encyclopedia of Gene and Genomes (KEGG) analysis also showed that these DEGs were enriched in pathways associated with the immune response, especially the intestinal immune network for IgA production, Cytokine-cytokine receptor interaction (Fig. 5e). This can explain how LP-H regulates the intestinal immune response, reduces inflammation, and maintains the intestinal barrier. We also identified the key hub proteins and interactions among these DEGs using STRING analysis. We found that the most significant interactions were between IL33 and IL1rl2, Tgfb1 and Ppara, Cxcr3 and Arl4c (Fig. 5f). These interactions are essential for the intestinal immune process, Especially the production of intestinal IgA. Overall, transcriptome analysis indicated that LP-H treatment modulates the immune response in intestinal tissues and facilitates recovery of the intestinal mechanical barrier in the constipation model.

## Discussion

Constipation has become very common and prevalent health condition in modern day society, characterized by slow colonic motility, delayed stool elimination, dry stools, and difficulty in defecation^19,20^. Constipation not only affects normal life but may also lead to intestinal diseases such as colitis and tumors^21^. The incidence of constipation is closely related to an imbalance in gut microbiota. Increasing evidence suggests that patients with constipation exhibit severe dysbiosis of the gut microbiota, and that reconstruction of the gut microbial community can effectively alleviate constipation symptoms^22,23^. Probiotics such as *Lactobacillus* are widely used in clinical practice for the treatment of colitis, abnormal fermentation in the intestines, and indigestion. Our previous research showed that *Lactobacillus* administration has a beneficial effect on the regulation of gut microbiota^18^. In this study, we investigated whether *Lactobacillus* can improve constipation by modulating gut microbiota. Our findings reveal that *Lactobacillus* improves bowel function by modulating gut microbiota, inhibiting the intestinal inflammatory environment, and maintaining the intestinal mucus barrier.

The most noticeable characteristics of the loperamide-induced constipation rat model are the quantity and water content of the feces and the intestinal transit rate^24^. In a pathological model, LP was found to increase body weight, fecal quantity, and water content in rats with constipation, while improving the intestinal transit rate, consistent with previous studies^20,25^. Furthermore, this improvement effect of LP was found to be dose-dependent, with higher concentrations of LP showing more significant improvement in constipation.

The integrity of the intestinal barrier relies on tight junctions between the intestinal epithelial cells and the mucus they secrete^26^. Zhang et al. found that constipation is associated with disruption of the integrity of the intestinal barrier leading to oxidative stress in colonic mucosa bacteria^27^. Consistent with this report, our results indicate that in constipated rats, the colonic mucosa become thinner, crypts become shorter, and goblet cells significantly decrease, all of which were subsequently found to be improved after intervention with LP. The intestinal mucus layer separates microbiota from epithelial cells, thereby preventing intestinal inflammation^28^. The main component of the mucus layer is the Muc2 protein secreted by goblet cells^26^. In our study, we found that LP increased the number of colonic goblet cells and the thickness of the mucus layer in constipated rats.

There are significant differences in the quantity and composition of intestinal microbiota between constipated patients and healthy individuals^29^. Some studies have shown that the relative abundance of *Bifidobacterium* and *Lactobacillus* is reduced in constipated patients, while the relative abundance of *Bacteroidetes*, *Fusobacterium*, and *Enterobacter* is enhanced^20,30^. Using 16 s rRNA sequencing technology, both α-diversity and β-diversity analyses in the current study revealed a significant increase in the abundance of the intestinal microbiota in constipated rats upon LP intervention. Through compositional analysis of different microbial groups, we found that the relative abundance of *Verrucomicrobiota* increased at the phylum level. At the family level, the relative abundance of *Akkermansiaceae* was found to increase, whereas the relative abundance of *Clostridiaceae* decreased. Further classification analysis revealed that at the genus level, the relative abundance of *Akkermansia* increased, whereas that of *Clostriium_sensu_stricto_1* decreased. At the species level, the relative abundance of *Akkermansia muciniphila* increased, whereas that of *uncultured_bacterium_g_Clostridium_sensu_stricto_1* decreased. *Akkermansia* is a bacterial species that can produce beneficial SCFAs and vitamins^31^. It improves intestinal permeability, reduces inflammation, and enhances the intestinal barrier function and metabolism by releasing anti-inflammatory vesicles and increasing mucus thickness^32^. This suggests that LP affects the quantity and composition of intestinal microbiota by promoting proliferation of beneficial bacteria and inhibiting proliferation of harmful bacteria.

RNA-seq data of rat colon tissues showed 87 differentially expressed genes (DEGs) among the three groups. GO analysis revealed that these genes were enriched for membrane and immune system processes. Further KEGG analysis indicated that these were enriched in pathways associated with the immune response, particularly the intestinal immune network for IgA production. PPI analysis also revealed changes in innate immune response. These results suggest that after LP intervention, the expression or distribution of receptors on the intestinal epithelial cell membrane that recognize pathogens may be altered, thereby regulating the innate immune response. However, further research is needed to elucidate the specific mechanisms by which *Lactobacillus* affects the intestinal inflammatory responses.

## Conclusion

This study focused on whether LP can alleviate constipation and modulate its underlying mechanisms of action. Based on previous findings, we hypothesized that LP promotes the colonization of *Akkermansia* and regulates the innate immune response, thereby improving constipation symptoms. The current results support this hypothesis and confirm that LP can improve constipation symptoms by modulating gut microbiota and regulating the immune response.

## Materials and methods

### Animals

All animal experiments in this study was approved by the experimental animal ethics committee of Jiangzhong Pharmaceutical Co., Ltd. (20,220,611). All procedures were carried out according to Guidelines for the Care and Use of Laboratory Animals and the Chinese Legislation on Laboratory Animals. The reporting in this study follows the recommendations in the ARRIVE guidelines. In this study, 6–8-week-old male SD rats from the Ke Ruisi Co., Ltd (Beijing, China) were raised in a standard environment (22.0 ± 1 °C, at a relative humidity of 45–55% under a 12-/12-h light/dark cycle). Feeding was strictly according to standard operating procedures (SOP) and the mice were free to eat and drink water. Vitals such as diet, body weight, and activity status of mice were monitored weekly.

### Regents and dosage information

LP was obtained from Jiangzhong Pharmaceutical Co., Ltd., Nanchang, China. Loperamide was purchased from Xian-Janssen Pharmaceutical Ltd. (Shaanxi, China).

### Constipation-model rats and experimental design

After one week of adaptive feeding, the rats were randomly separated into four groups: (1) control group, (2) loperamide-induced constipation model group (model group), (3) loperamide-induced constipation model + low-dose LP treatment group (LP-L group), and (4) loperamide-induced constipation model + high-dose LP treatment group (LP-H group). Rats in the control group were fed standard chow without any treatment. With the exception of the control group, constipation was induced other groups by oral administration of 10 mg/kg loperamide once a day to establish the constipation model, and the rats were given continuous gavage for 2 week. Low-dose LP (420 mg/kg) and high-dose LP (820 mg/kg) were then administered daily for ten days starting from the fourth day after loperamide induction. During the trial, the general physiological state and fecal condition of the rats were recorded daily.

### 24-h defecation and fecal water content

At the end of the last treatment, rats were housed individually in cages to collect feces for 24 h, and the fecal number and fecal weight were recorded subsequently. The fecal water content was calculated after drying the feces in a desiccator at 60℃ for 12 h, according to the equation: (wet weight − dry weight)/wet weight × 100%.

### Small intestinal propulsion trial

To investigate intestinal motility, rats in each group were gavaged with Evans blue paste meal 30 min after the last treatment. After 20 min of the meal, the rats were euthanized by Carbon dioxide anesthesia, the abdominal cavities were dissected, and the intestines from the pylorus to the ileocecal portion were extracted. The intestinal transit rates were calculated using the formula: distance traveled by activated Evans blue in the intestine (cm)/full length of the small intestine (cm) × 100%.

### 16S rRNA gene sequencing

DNA was extracted from the colon material, followed by 16S rRNA gene amplification, purification, library preparation, and paired-end sequencing on the Illumina MiSeq platform. Detailed protocols are available in the literature^19^. The raw reads were pre-processed using the MICCA pipeline (v.1.5) available at http://www.micca.org. Trimmed and quality-filtered reads for both forward and reverse primers were obtained using Micca trim and Micca filter, respectively. De novo clustering and chimera filtering were performed using Micca otu with a pairwise identity threshold of 97% for sequence clustering. Taxonomic classification of representative sequences was performed using the Micca classification with the RDP classifier. Multiple sequence alignment (MSA) of the 16S rRNA gene sequences was performed using the Nearest Alignment Space Termination (NAST) algorithm within the micca msa, with template alignment clustered at 97% similarity based on the Greengenes database. To reduce heterogeneity due to sampling, rarefaction of samples was performed at the depth of the least abundant sample using Micca tablets. Alpha (within-sample richness) and diversity (between-sample dissimilarity) estimates were calculated using the Phyloseq R package vegan with 999 permutations. Linear discriminant effect size analysis (LEfSe) was employed to identify microbial taxa that were most likely to explain the differences between classes. Random Forest analysis of the 16S rRNA gene sequencing data was conducted using the Random Forest R package and model significance was assessed through permutation tests with 1000 permutations.

### Histopathological examination

Distal colon tissues were fixed with 4% polyformaldehyde solution overnight, embedded in paraffin, and divided into 5 μm thick slices. The slices were then subjected to hematoxylin and eosin (H&E) staining and immunofluorescent staining and were imaged using Zeiss Axioscope light microscope . The images obtained were then analyzed using image J software.

### RNA sequencing

Rat colons were harvested and RNA samples were isolated as previously described^20^. RNA samples were prepared according to the manufacturer’s recommended protocol, indexed, pooled, and sequenced using an Illumina Nova-Seq 6000. The reference genome and gene model annotation files were downloaded from the Ensemble database. Fragments per kilobase of transcript per million (FPKM) mapped reads HtSeq count was used to count the read numbers mapped to each gene. FPKM of each gene was calculated based on the length of the gene and the read count mapped to this gene. Differential expression of all groups was calculated using the DESeq2 R package (1.16.1). Differentially expressed genes (DEGs) were defined as *p* value < 0.05 and FC > 2. Gene Ontology (GO) enrichment analysis of DEGs was performed by the topGO R package with correction applied for gene length bias. Kyoto Encyclopedia of Genes and Genomes (KEGG) pathway enrichment analysis of DEGs was performed using the Cluster Profiler R package^33^. Protein–protein interaction (PPI) analysis of DEGs was performed using the STRING database.

### Statistical analysis

All data are presented as mean ± SD for each group. After normality testing, differences between groups were analyzed using one-way ANOVA analysis with Dunnett’s post hoc test for normally distributed data. Non-normally distributed data were analyzed using the Kruskal–Wallis non-parametric method. All regular plots were generated using GraphPad Prism 8. *p*-values less than 0.05 were considered significant (*p < 0.05, **p < 0.01).

### Ethical declarations

All animal experiments in this study was approved by the experimental animal ethics committee of Jiangzhong Pharmaceutical Co., Ltd. (No. 20220611). All procedures were carried out according to Guidelines for the Care and Use of Laboratory Animals and the Chinese Legislation on Laboratory Animals. The reporting in this study follows the recommendations in the ARRIVE guidelines.

## Data Availability

All the data that were used to support the findings of this study are included within the article. 16 s RNA-seq dates were deposited to the SRA at https://dataview.ncbi.nlm.nih.gov/object/PRJNA1041582?reviewer=m939qsidptb8ropm3ernspiecg. (BioProject ID: PRJNA1041582). RNA-seq dates of colon were deposited to the SRA at https://dataview.ncbi.nlm.nih.gov/object/PRJNA1041297?reviewer=2klu441vrpjdapu1q216ghbcc7. (BioProject ID: PRJNA1041297). Data generated and analyzed during this period can be obtained from the corresponding author upon reasonable request.
